# Supplementary material for: Biallelic JAK1 mutations in immunodeficient patient with mycobacterial infection
Source: Nat Commun. 2016 Dec 23;7:13992. doi: 10.1038/ncomms13992 (PMC5196432; doi:10.1038/ncomms13992)

SUPPLEMENTARY INFORMATION

Supplementary Table 1. Rare coding homozygous variants found by exome sequencing

Chr	Position	Ref/Obs	Ensembl<br>Exon, cDNA	Gene (name)	Amino acid	PolyPhen-2 prediction (score)
1	65,307,194	G/A	ENSG00000162434<br>Exon 18, c.C2494T	JAK1 (Janus kinase 1)	p.P832S	Probably Damaging (0.997)
1	65,310,490	G/A	ENSG00000162434<br>Exon 16, c.C2198T	JAK1 (Janus kinase 1)	p.P733L	Probably Damaging (0.999)
7	73,935,550	C/T	ENSG00000006704<br>Exon 7, c.C929T	GTF2IRD1 (GTF2I repeat domain containing 1)	p.T310I	Benign (0.003)
9	75,780,123	G/A	ENSG00000135046<br>Exon 8, c.G704A	ANXA1 (annexin A1)	p.R235K	Benign (0)
14	39,796,197	A/G	ENSG00000150527<br>Exon 20, c.A1802G	CTAGE5 (CTAGE family, member 5)	p.Q601R	Benign (0.105)

Supplementary Table 2. Oligonucleotides used for cloning, mutagenesis and qPCR

Primer name	Sequence 5'- 3'	Application
JAK1A2	TTTGGTACCATGCAGTATCTAAATATAAAAGAGG	Cloning JAK1 into pcDNA3.1
JAK1B	TTTCTCGAGTTATTTTAAAAGTGCTTCAAATCC	
J1E	CCCTGATCAATGCAGTATCTAAATATAAAAGAGG	Cloning JAK1 into pHR-UbEm
J1F	CCCGCGGCCGCTTATTTTAAAAGTGCTTCAAATCC	
J1A	CCCGCTGCATGAACTATGACTCCAATCAGAGGCCTTTCTTCCG	Quickchange P832S
J1B	CGGAAGAAAGGCCTCTGATTGAGGTCATAGTTCATGCAGCGGG	
J2A	GGGCATCGACAGTGAGTGTGGCCTATTCATCAAGCTCAGTGACCCCGG	Quickchange P733L
J2B	CCGGGGTCACTGAGCTTGATGAATAGGCCACACTCACTGTCGTCGATGCCC	
JAK1_KE_anti	TCAGGCTTCAGAGATTCAACAGCCACCTGCTCC	Quickchange K908E
JAK1_KE_sense	GGAGCAGGTGGCTGTTGAATCTCTGAAGCCTGA	
K_DEL_Fw	ATACGTCTCACTTCAAATCTAGAGGGCCCTTCGA	Golden Gate Assembly (JAK1$^{Kin\Delta}$)
K_DEL_Rv	CGCCGTCTCGGAAGCGCTTTTCAAAATGT	
PK_DEL_Fw	GGACGTCTCAGGATGTTTCAGAAAAAAAACCAGC	Golden Gate Assembly (JAK1$^{\Psi Kin\Delta}$)
PK_DEL_Rv	ACACGTCTCGATCCTTCTTGAGGATCCGA	
JAK1-Kpn-Fw	AAGCTTGGTACCATGCAGTATC	Cloning JAK1 in pcDNA6A-myc-His
JAK1-Xba-Rv	GCTCTAGATTTTAAAAGTGCTTCAAATCC	
XBAI_sfGFP_Fw	gctctagaGTGAGCAAGGGCGAGGAGCT	Cloning sfGFP in JAK1-pcDNA6A
XBAI_sfGFP_Rv	gctctagattaCTTGTACAGCTCGTCCAT	
MX1_F	TCACCAGAGAATAACAGAGG	qPCR
MX1_R	GGCATTAACTTTATCTATCAGGAA	
OAS1_F	GATTCTGCTGGCTGAAAG	
OAS1_R	ATGTGTTCCAATGTAACCATAT	
IRF9_F	AAGTTCCAGGTAACACTGA	
IRF9_R	CTGCTCCATCTTCACTGT	
IRF1_F	CAGAGAAAAGAAAGAAAGT	
IRF1_R	CATCAGAGAAGGTATCAG	
IFIT1_F	GCTCCAGACTATCCTTGACCT	
IFIT1_R	CCACAAGACAGAATAGCCAGAT	
TAP1_F	CTGAGGTGCTGGGTGATG	
TAP1_R	GTGTACTTATCCTGGATGATGCC	
IFITM1_F	ATGAATCCAATGGTCATGAGGA	
IFITM1_R	CTTCATAGCATTCGCCTACTCC	

ISG15_F	GCCTTCAGCTCTGACACC
ISG15_R	CGAACTCATCTTTGCCAGTACA
IF16_F	GTAGCACAAGAAAAGCGATACC
IF16_R	CTGCTGTGCCCATCTATCAG
IFIT3_F	CACTGTCTTCCTTGAATAAGTTCC
IFIT3_R	AGAACAAATCAGCCTGGTCAC
ADAR_F	CACTTTCTTGCTTCCAGCTTC
ADAR_R	CTGTGAGTTCAACATGATAGAGC
IFI30_F	GAGCTGCAGGCATAGTGG
IFI30_R	CAGCATGGAGAAGAGGAGTG
EIF2AK2_F	CCAAATCCACCTGAGCCAAT
EIF2AK2_R	CCAGATTTGACCTTCCTGACA
HPRT_F	TGCTCGAGATGTGATGAAGG
HPRT_R	TCCCCTGTTGACTGGTCATT

Supplementary Figures

Supplementary Fig. 1. Janus kinases protein sequence alignment

(**a**) Protein sequence of human Janus kinases. (**b**) Protein sequence of JAK1 in various species.

a

```
JAK1           AREG-IDSECGPFIKLSDPGIPITVLSRQECIERIPWIAPECVEDSKN-LSVAADKWSFG 780
TYK2           ARLG-LAEGTSPFIKLSDPGVGLGALSREERVERIPWLAPECLPGGANSLSTAMDKWGFG 801
JAK2           IREEDRKTGNPPFIKLSDPGISITVLPKDILQERIPWVPPECIENPKN-LNLATDKWSFG 740
JAK3           AREG--ADGSPPFIKLSDPGVSPAVLSLEMLTDRIPWVAPECLREAQT-LSLEADKWGFG 712
                *         *********:   .*. :   :****:.***:    . *.   ***.**

JAK1           TTLWEICYNGEIPLKDKTLIEKERFYESRCRPVTPSCKELADLMTRCMNYDPNQRPFFRA 840
TYK2           ATLLEICFDGEAPLQSRSPSEKEHFYQRQHRLPEPSCPQLATLTSQCLTYEPTQRPSFRT 861
JAK2           TTLWEICSGGDKPLSALDSQRKLQFYEDRHQLPAPKWAELANLINNCMDYEPDFRPSFRA 800
JAK3           ATVWEVFSGVTMPISALDPAKKLQFYEDRQQLPAPKWTELALLIQQCMAYEPVQRPSFRA 772
               :*: *:  .   *:.     .* :**: : :   *.  :** *  .*: *:*  ** **:
```

b

```
Human          S-ECGPFIKLSDPGIPITVLSRQECIERIPWIAPECVEDSKNLSVAADKWSFGTTLWEIC 787
Chimpanzee     S-ECGPFIKLSDPGIPITVLSRQECIERIPWIAPECVEDSKNLSVAADKWSFGTTLWEIC 787
Dog            S-ECGPFIKLSDPGIPITVLSRQECIERIPWIAPECVEDSKNLSVAADKWSFGTTLWEIC 786
Armadillo      S-ECGPFIKLSDPGIPITVLSRQECIERIPWIAPECVEDSRKLSVAADKWSFGTTLWEIC 785
Mouse          S-DIGPFIKLSDPGIPVSVLTRQECIERIPWIAPECVEDSKNLSVAADKWSFGTTLWEIC 786
Chicken        T-EYGPFIKLSDPGIPITVLSRQECVERIPWIAPECVEDSKNLSIAADKWSFGTTLWEIC 781
Xenopus        N-DCGPFIKLSDPGIPITVLTRQERVERIPWIAPECVEDSRVLSVAADKWSFGTTLWEIC 775
Zebrafish      G-EGGPFIKLSDPGIPITVLSREECVDRIPWIAPECVKDTANLSIAADKWSFGTTLWEIC 786
Cave_fish      S-EGGPFIKLSDPGIPITVLTREECVDRIPWIAPECVQDTANLSVAADKWGFGTTLWEIC 795
Fugu           IDEGGPFIKLSDPGIPITVLTREECVHRIPWIAPECVKNMSSLSVAADKWGFGTTLWEIC 801
Cod            VSEGGPFIKLSDPGIPITVLTREECVGRIPWIAPECIKTVNNLSVAADKWGFGTTLWEIC 805
                 : ************::**:*:* : *********::    **:*****.*********

Human          YNGEIPLKDKTLIE-KERFYESRCRPVTPSCKELADLMTRCMNYDPNQRPFFRAIMRDIN 846
Chimpanzee     YNGEIPLKDKTLIE-KERFYESRCRPVTPSCKELADLMTRCMNYDPNQRPFFRAIMRDIN 846
Dog            YNGEIPLKDKTLIE-KERFYESRCRPVTPSCKELADLMTRCMNYDPNQRPFFRAIMRDIN 845
Armadillo      YNGEIPLKDKTLIE-KERFYESRCRPVTPSCKELADLMTRCMNYDPNQRPFFRAIMRDIN 844
Mouse          YNGEIPLKDKTLIE-KERFYESRCRPVTPSCKELADLMTRCMNYDPNQRPFFRAIMRDIN 845
Chicken        YNGETPLKDKTLAE-KERFYEGHFMLITPSCKELADLMKQCMNYDPHQRPFFRAIMRDIN 840
Xenopus        FNGEVPLKDRTLAE-KERFYGGCFMLVAPSCKELADLINQCMNYDPLRRPFFRAIMREIN 834
Zebrafish      YNGEIPLKDKKLTE-KERFYAAQCQLATPDCDELAKLMTHCMTYDPRQRLFFRAIVRDIV 845
Cave_fish      YNGETPLKEKKLTE-KERFYAAQCQLATPDCQELAKLMTHCMNYDPHQRPFFRAIVRDID 854
Fugu           YDGEVPLKEKKLTE-KERFYETKCQLATPDCKELAELMTHCMNYDPKKRPFFRAIVRDID 860
Cod            YNGEVPLKDKMLTEQKEQFYSAECQLATPDCEELAQLMTHCMNYDPKMRPFFRAIVRDMD 865
               ::** ***:: * * **:**       :*.*.***.*:.:**.***  * *****:*::
```

Supplementary Fig. 2. JAK1 protein expression

(**a**) Level of expression of the endogenous JAK1 protein in primary fibroblasts derived from the patient (P) or two healthy control (C1, C2), assayed by immunoblotting. Three biological replicates and quantification by densitometry are shown. Fold change of band densitometry is indicated (numbers below bands and bar graphs). Graphs show mean values ± s.d. (**b**) Immunoblotting of protein extracts from HEK-293T cells transiently expressing wild type JAK1^{WT}, patient-derived mutant JAK1$^{P733L/P832S}$ or and the kinase-dead mutant JAK1^{K908E}. Two tagged versions (–myc or –sfGFP) are shown. Representative of three independent experiments.

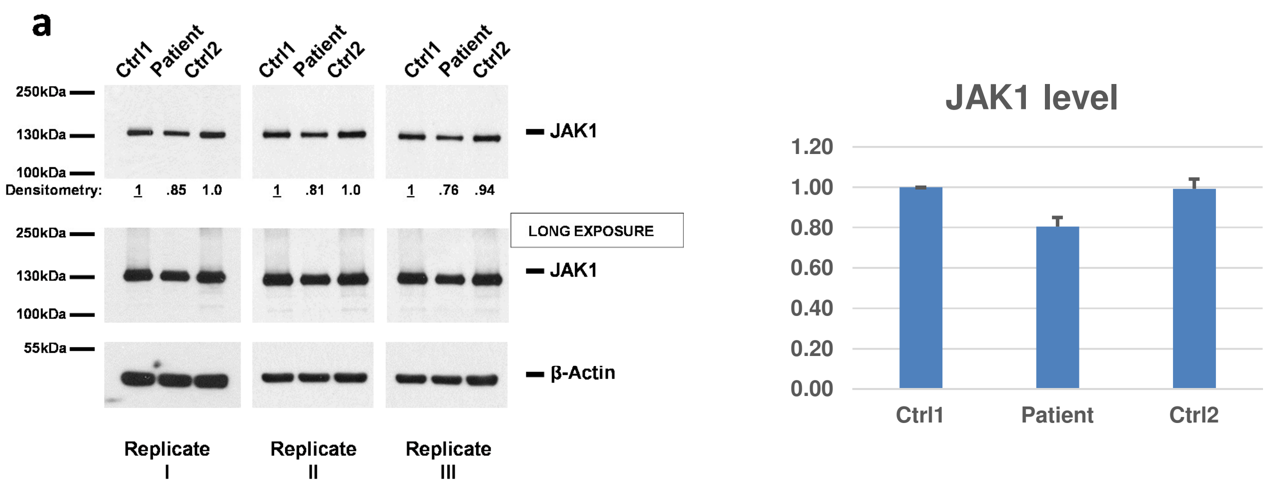

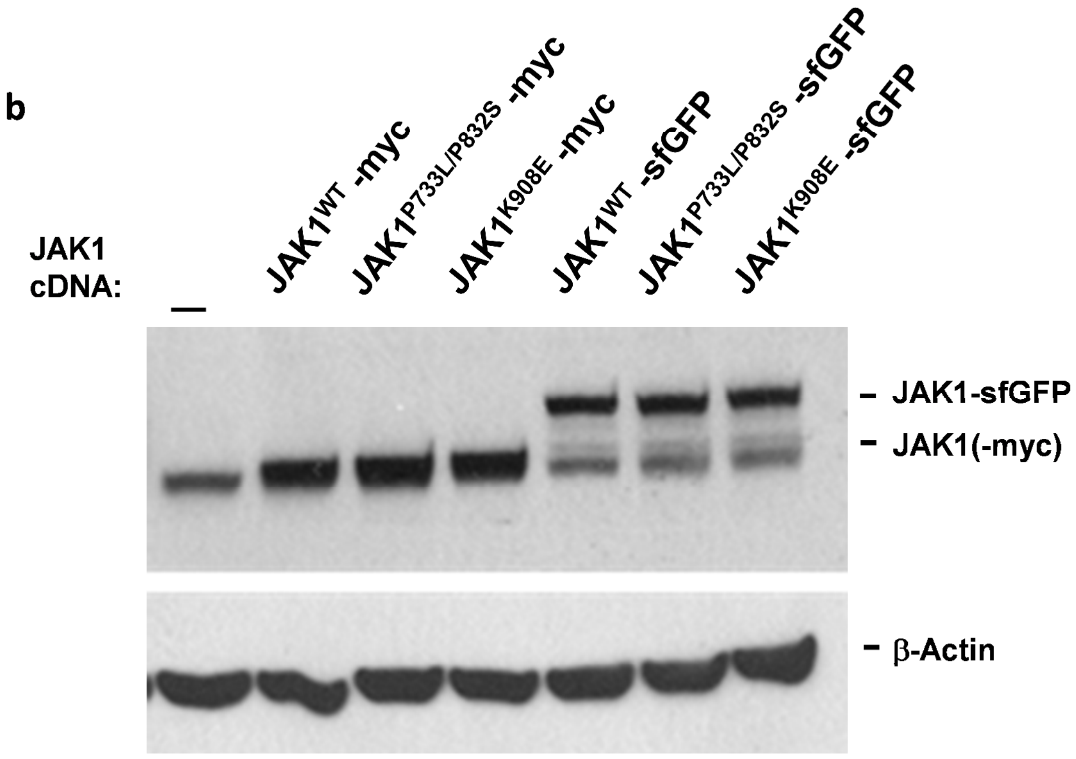

Supplementary Fig. 3. **Patient's fibroblasts show reduced induction of gene expression after IFN-α and IFN-γ stimulation.** Cells were stimulated with IFN-α for 15 hours (**a**) or IFN-γ for 8 hours (**b**). mRNA fold change is shown relative to the untreated control fibroblasts. * $P < 0.05$, ** $P < 0.005$, *** $P < 0.0005$. Graphs show mean values ± s.d.

a

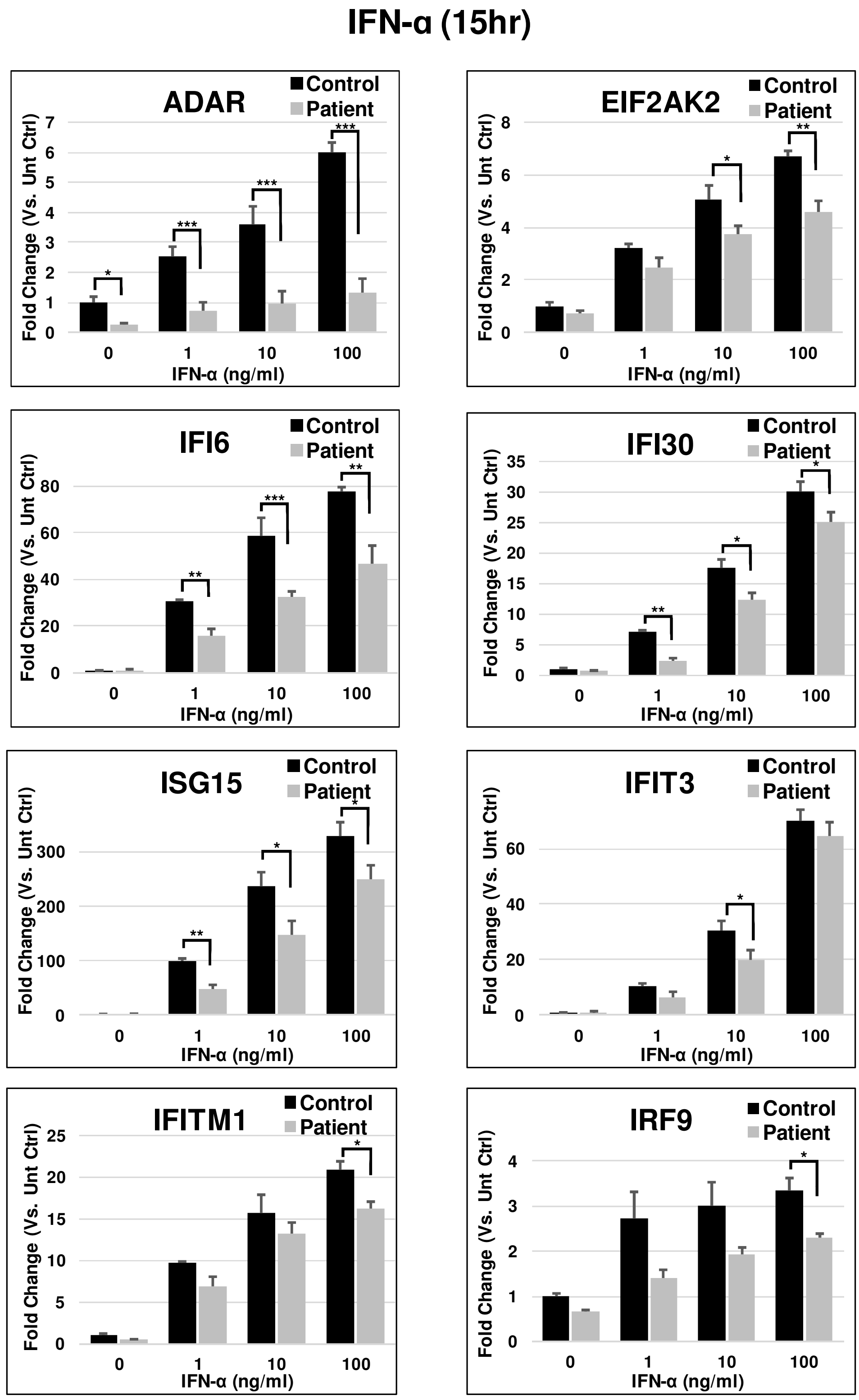

Supplementary Fig. 3.

b

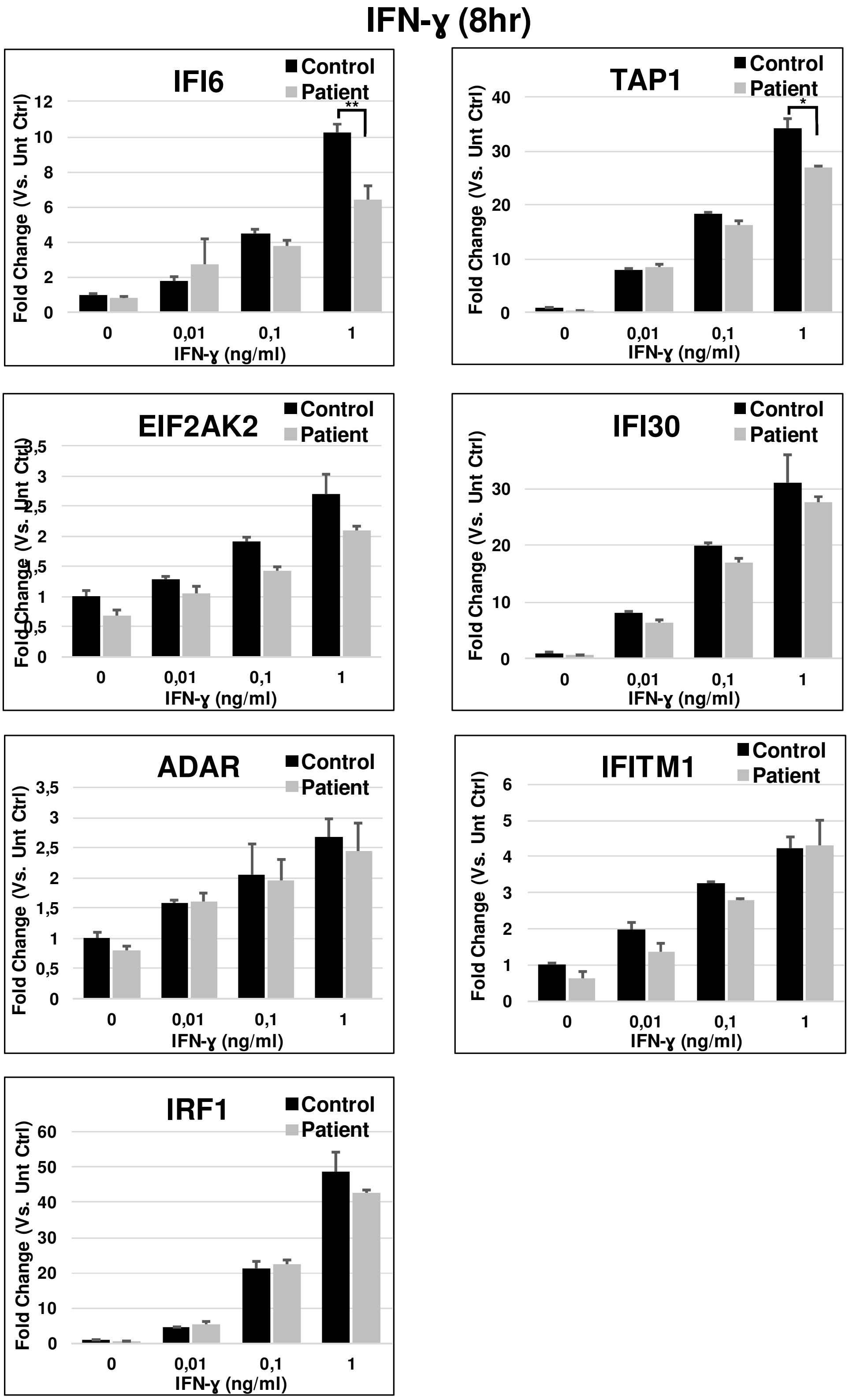

Supplementary Fig. 4. JAK1 constructs
Schematic presentation of the JAK1 constructs expressed in the JAK1-deficient Flp-In U4C cells.

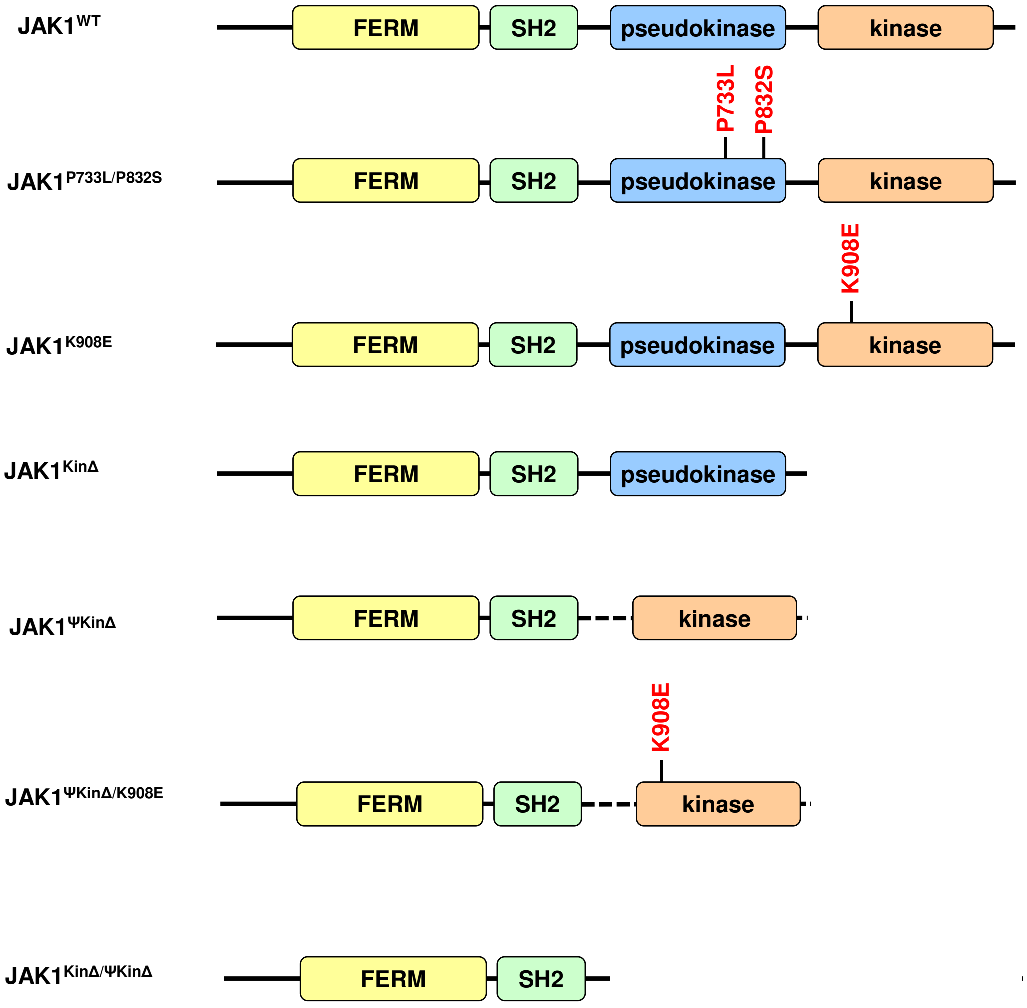

Supplementary Fig. 5. Subcellular localization of the sfGFP-fused JAK1 proteins stably expressed in the U4C cell clones

Confocal microscopy showing cells of the stable clones of the JAK1-deficient Flp-In U4C expressing sfGFP-fused $JAK1^{WT}$, $JAK1^{P733L/P832S}$, $JAK1^{K908E}$, $JAK1^{Kin\Delta}$, $JAK1^{\Psi Kin\Delta}$, $JAK1^{\Psi Kin\Delta/K908E}$ or $JAK1^{Kin\Delta/\Psi Kin\Delta}$ constructs.

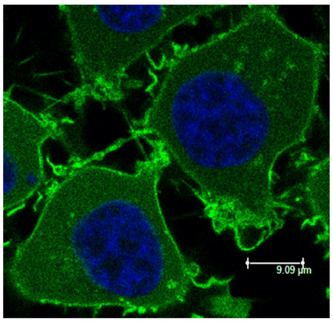

$JAK1^{WT}$

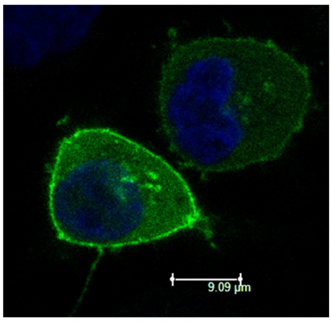

$JAK1^{P733L/P832S}$

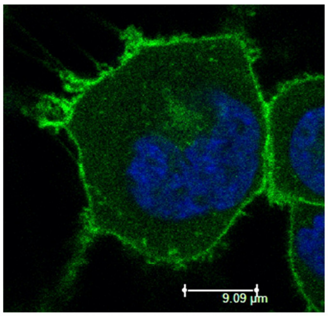

$JAK1^{Kin\Delta}$

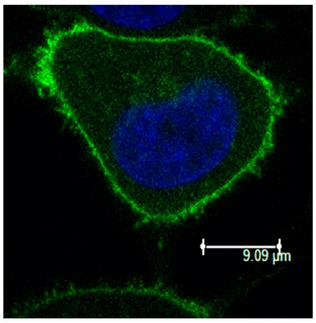

$JAK1^{\Psi Kin\Delta}$

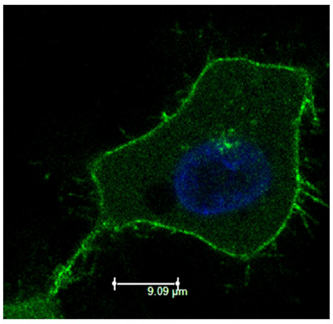

$JAK1^{K908E}$

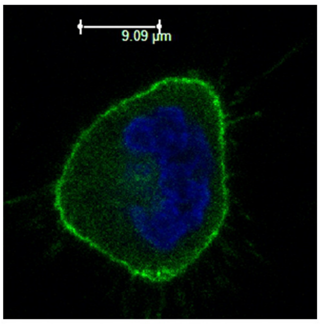

$JAK1^{\Psi Kin\Delta/K908E}$

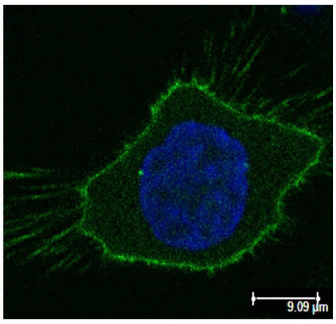

$JAK1^{Kin\Delta/\Psi Kin\Delta}$

Supplementary Fig. 6.

Densitometry quantification of western blots shown in Fig. 3. Graphs show mean values ± s.d.

a

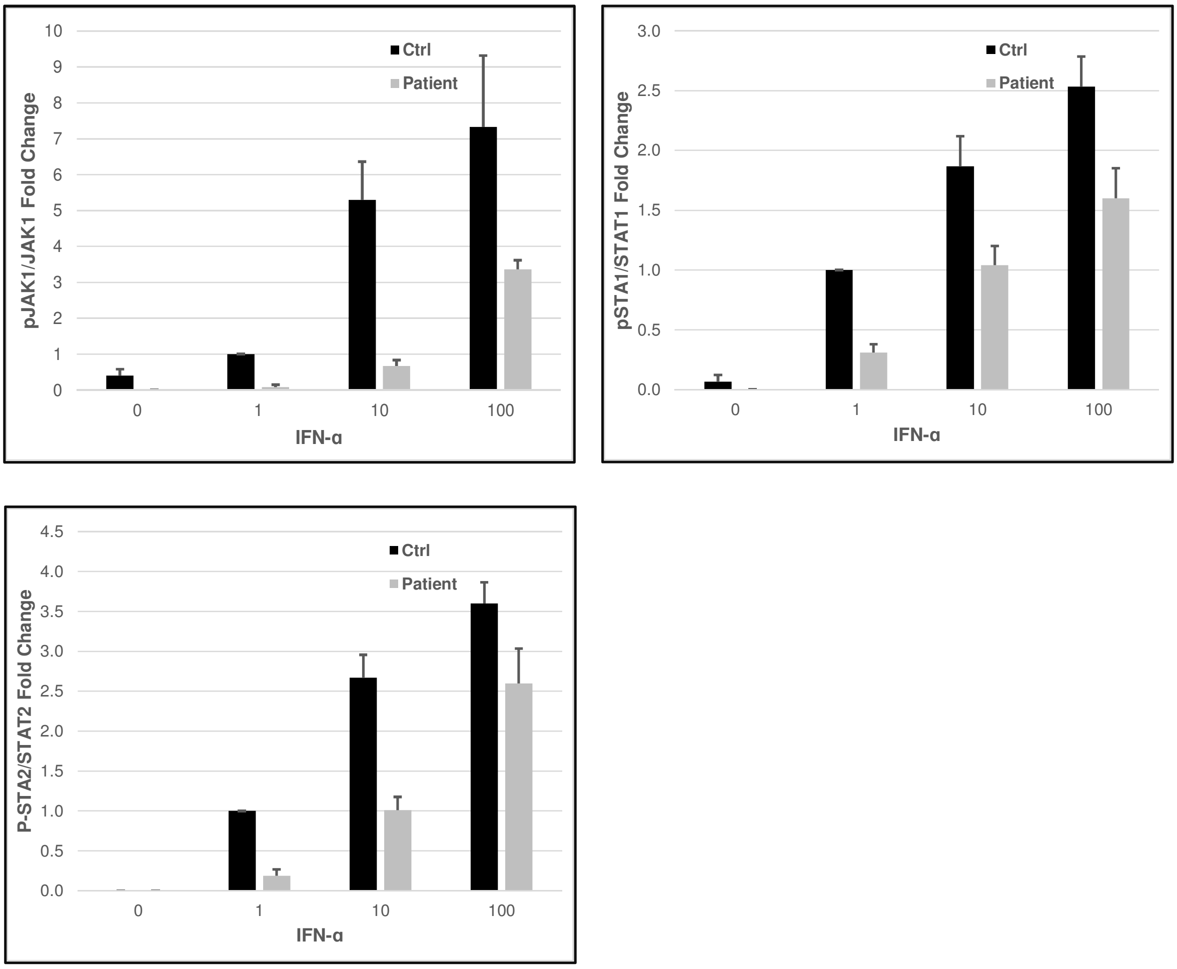

b

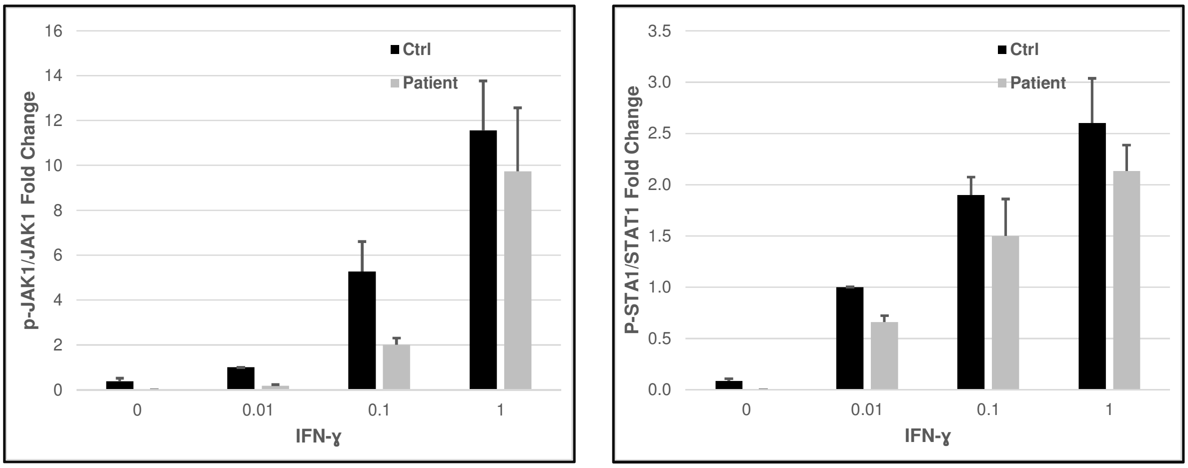

Supplementary Fig. 7.
Densitometry quantification of western blots shown in Fig. 5. Graphs show mean values ± s.d.

a

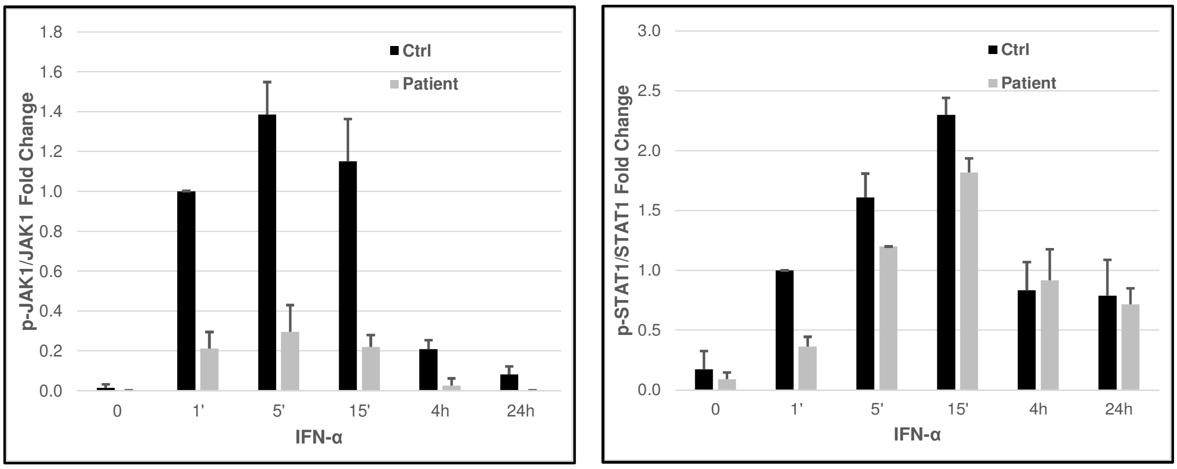

b

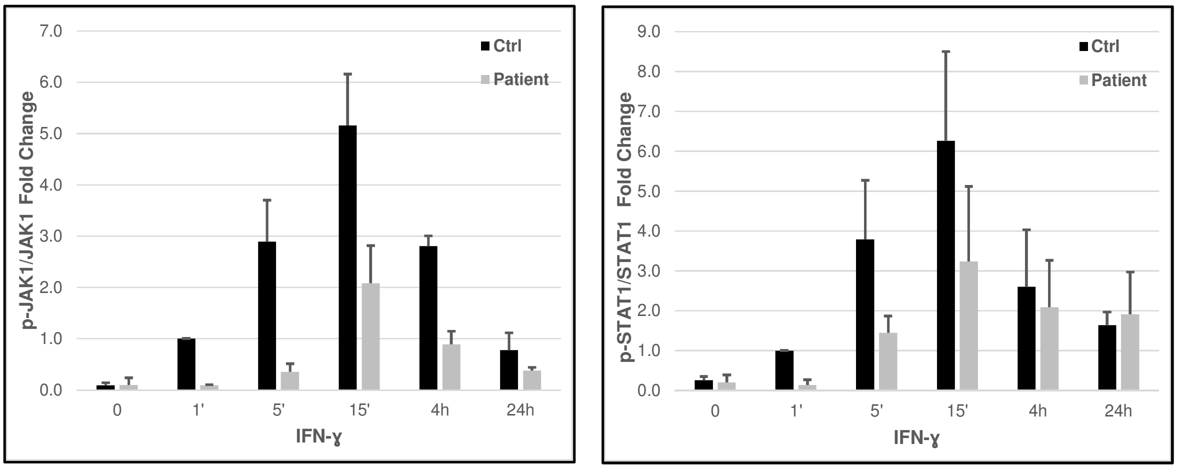

Supplementary Fig. 8.
Densitometry quantification of western blots shown in Fig. 6. Graphs show mean values ± s.d.

a

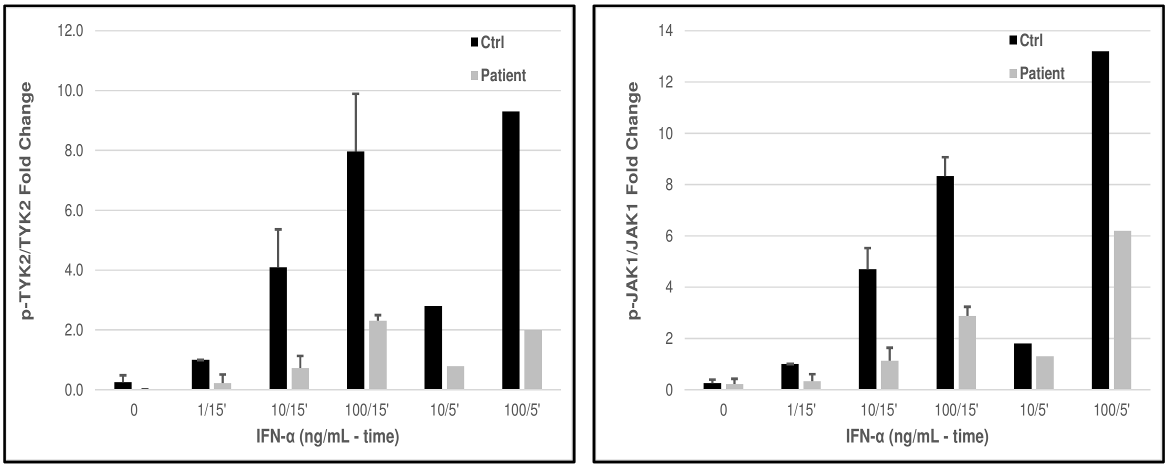

b

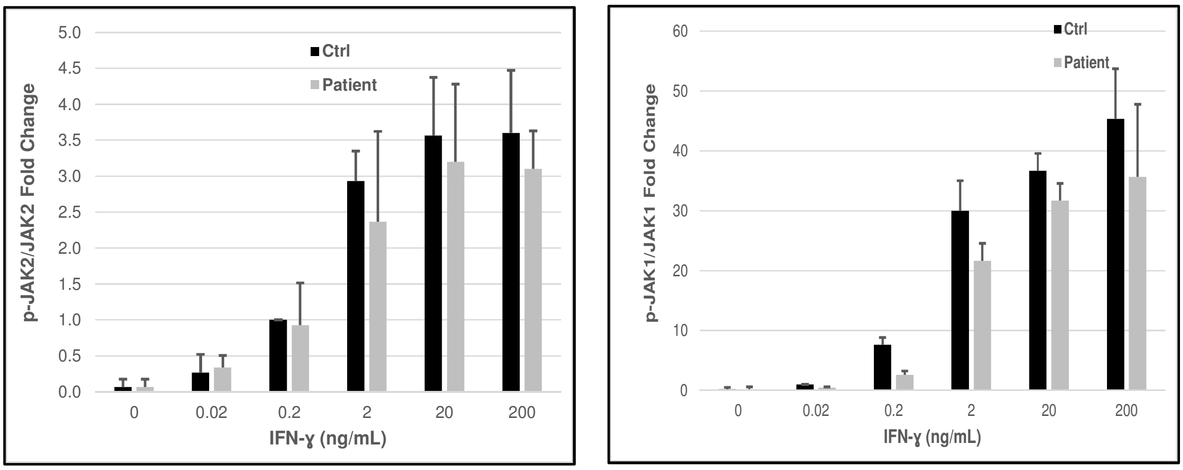

Supplementary Fig. 9.
Densitometry quantification of western blots shown in Fig. 7. Graphs show mean values ± s.d.

a

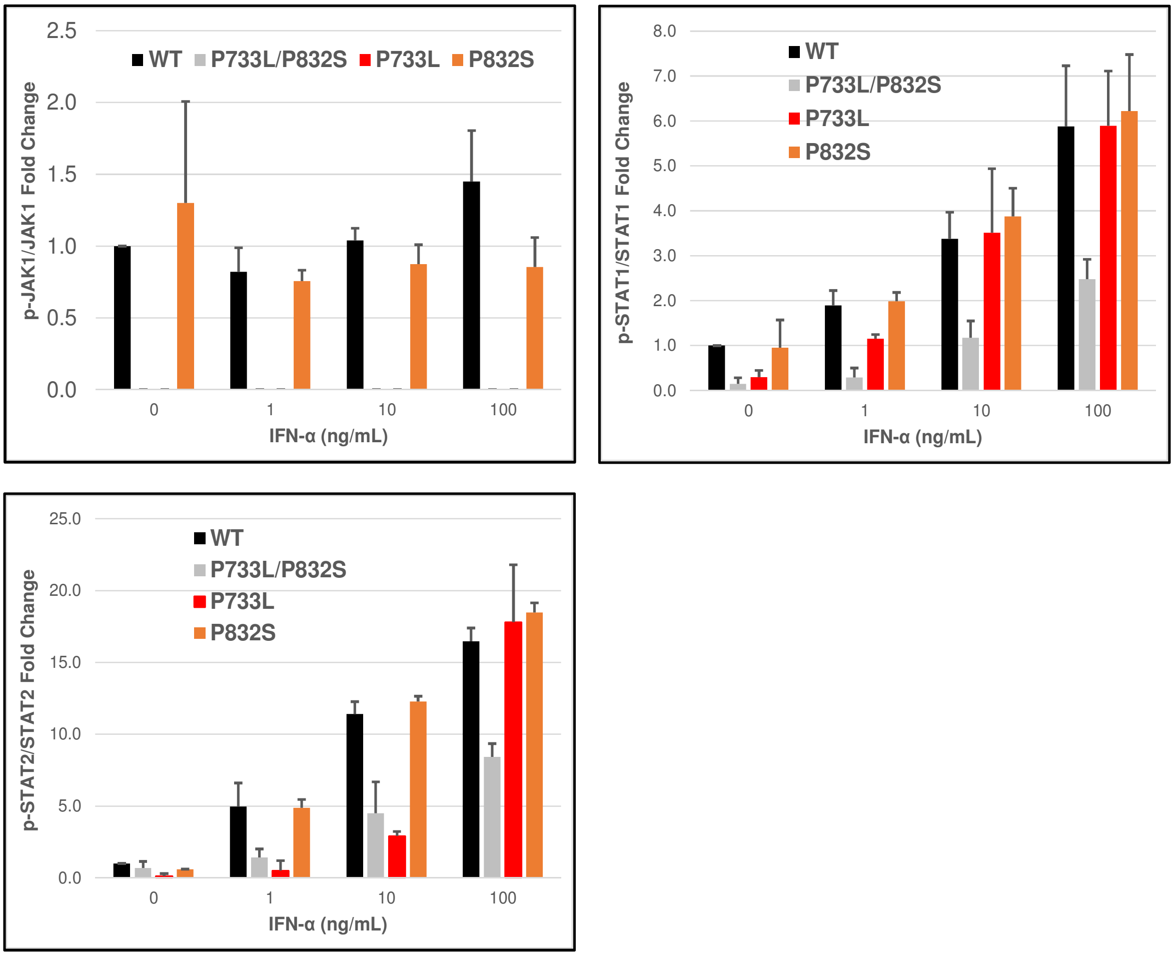

b

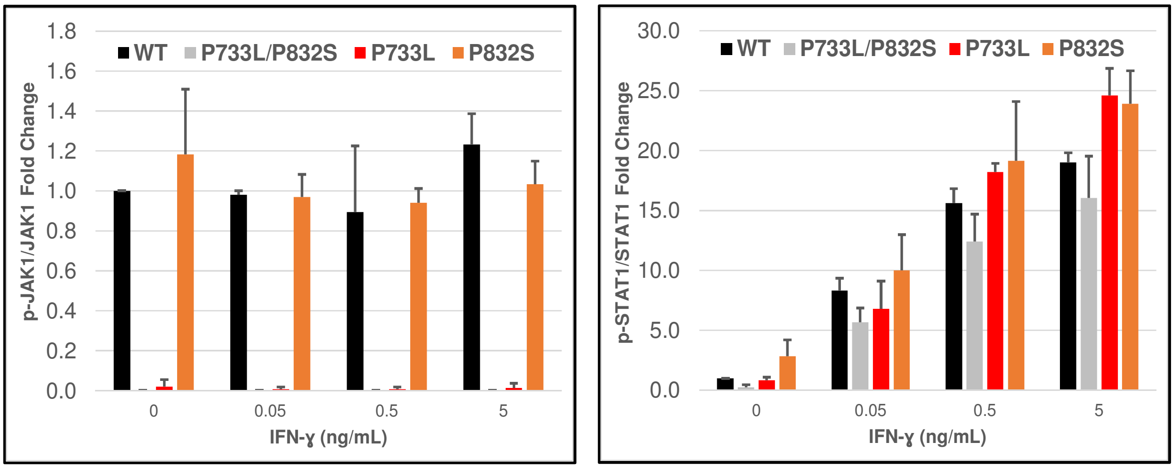

Supplementary Fig. 10.
Densitometry quantification of western blots shown in Fig. 8. Graphs show mean values ± s.d.

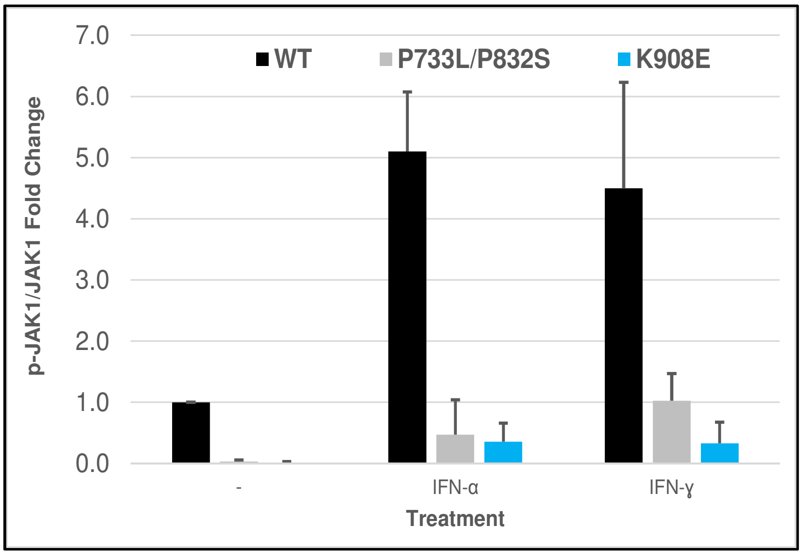

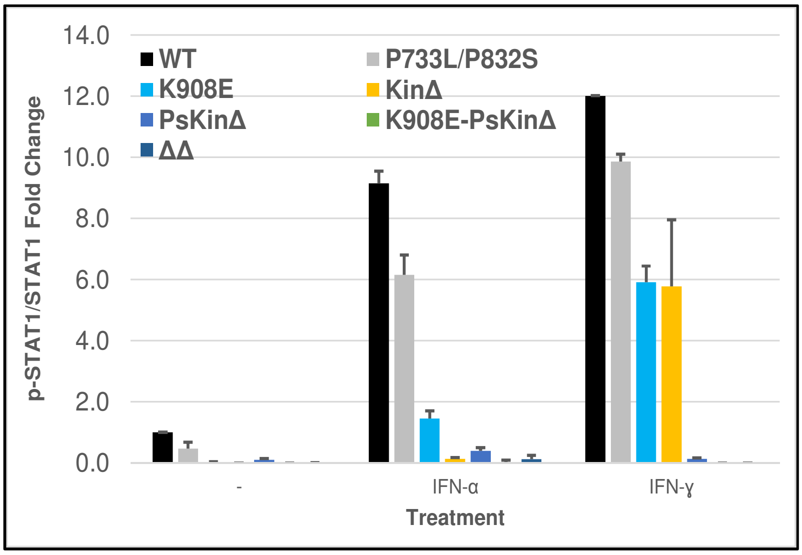

Supplementary Fig. 11. Uncropped scans of western blots shown in Figs 3, 5, 6, 7, 8 and Supplementary Fig. 2.

UNCROPPED SCANS – Fig. 3A

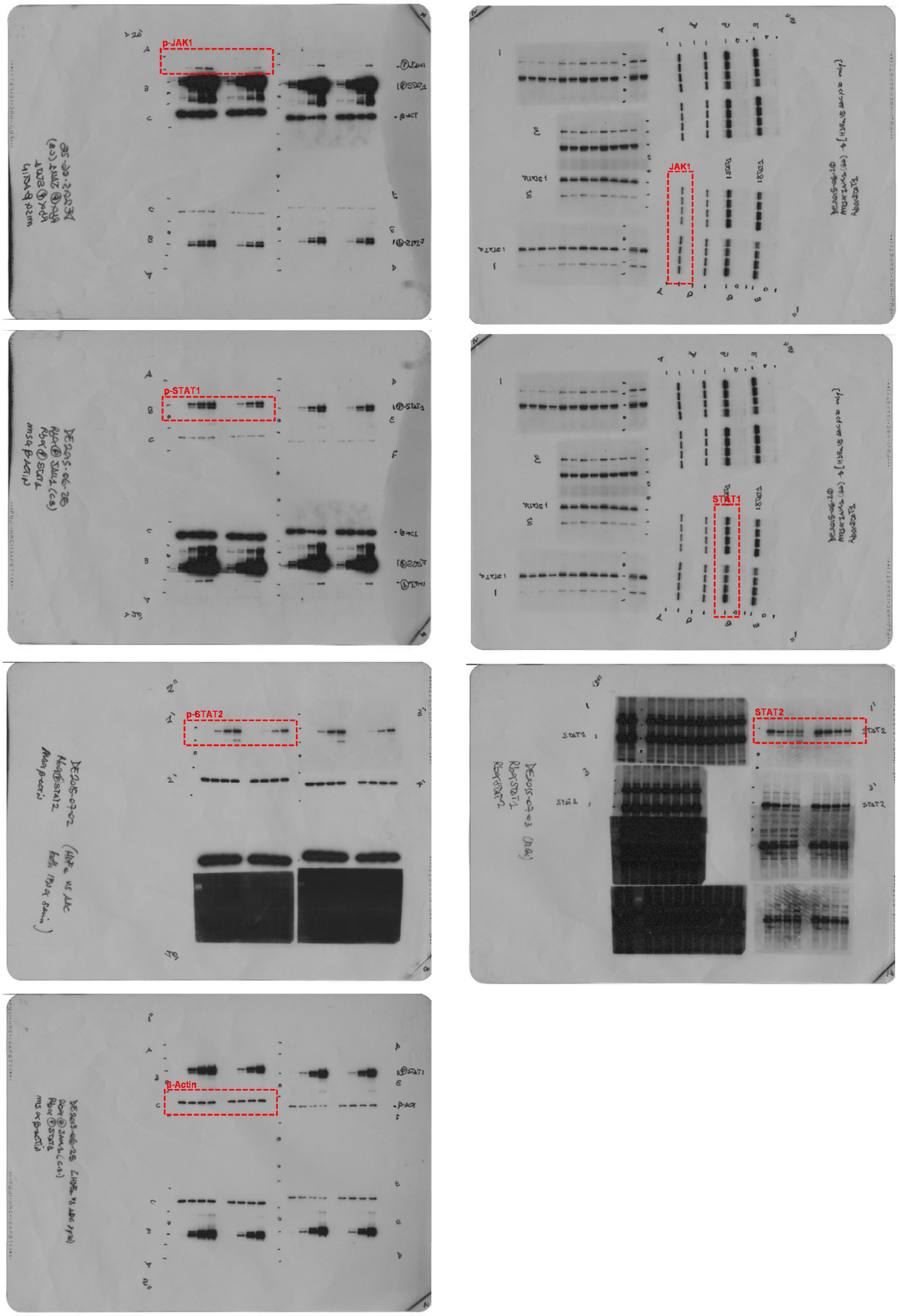

UNCROPPED SCANS – Fig. 3B

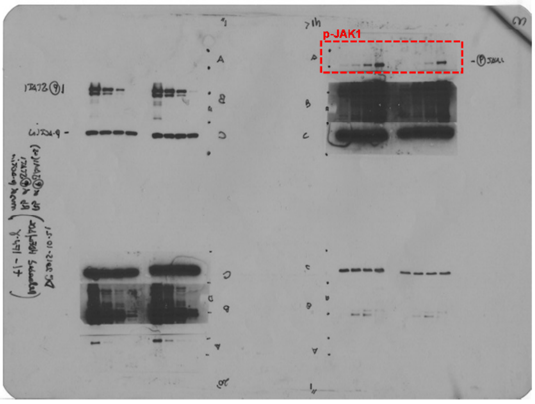

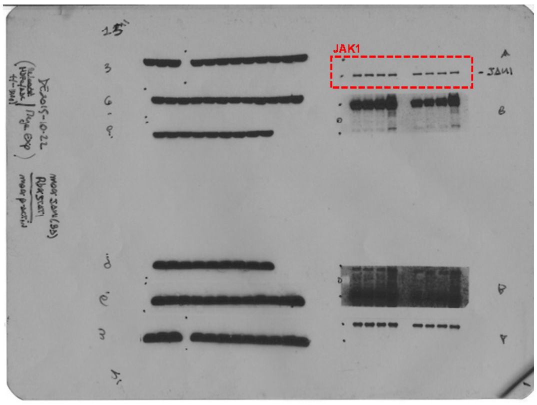

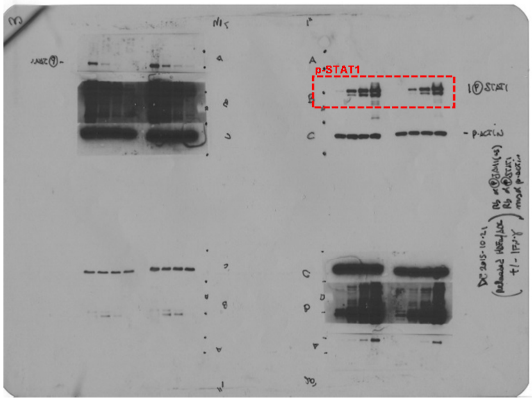

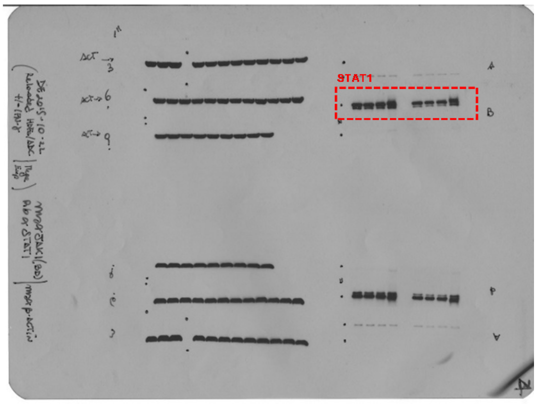

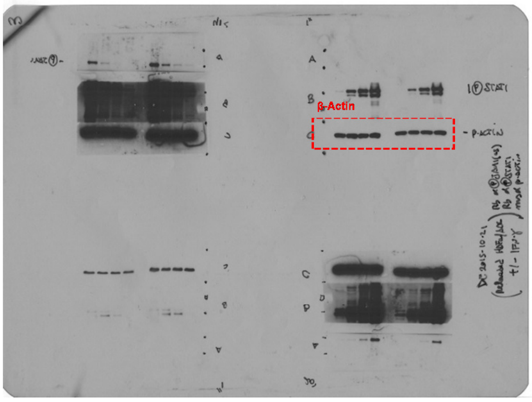

UNCROPPED SCANS – Fig. 5A

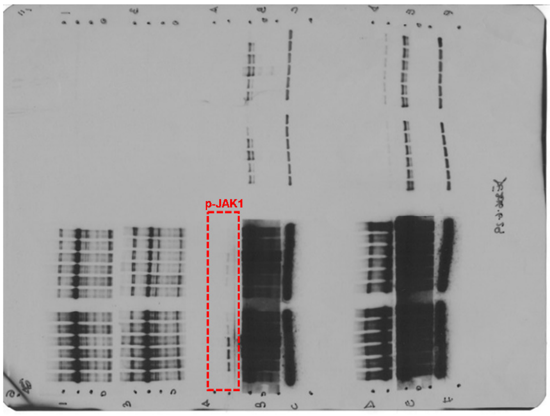

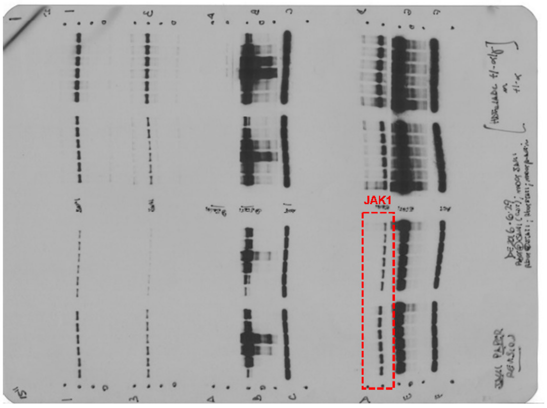

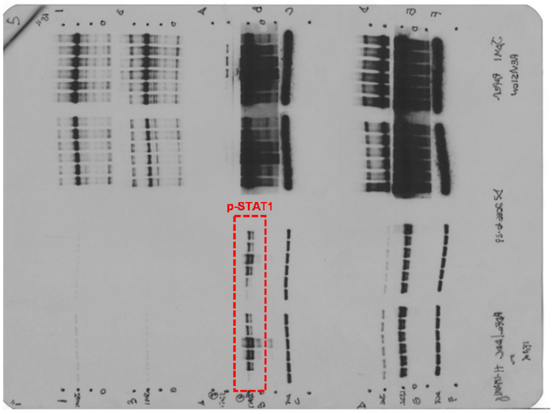

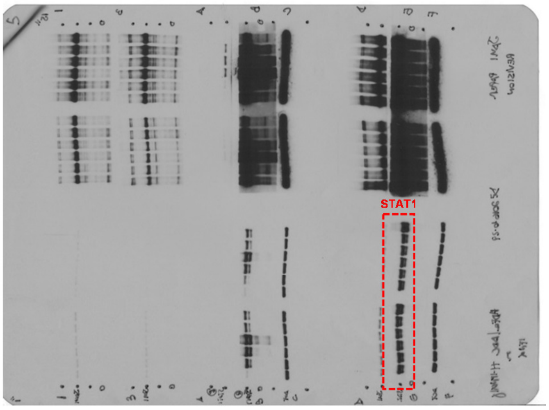

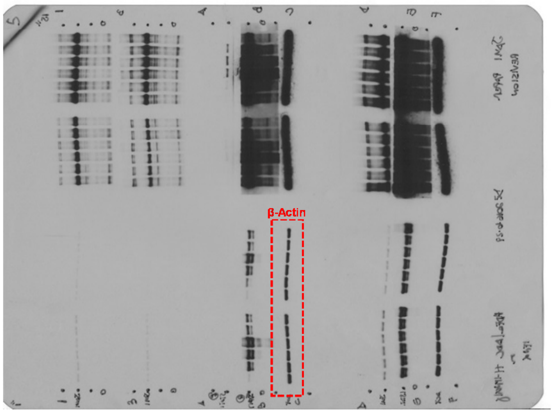

UNCROPPED SCANS – Fig. 5B

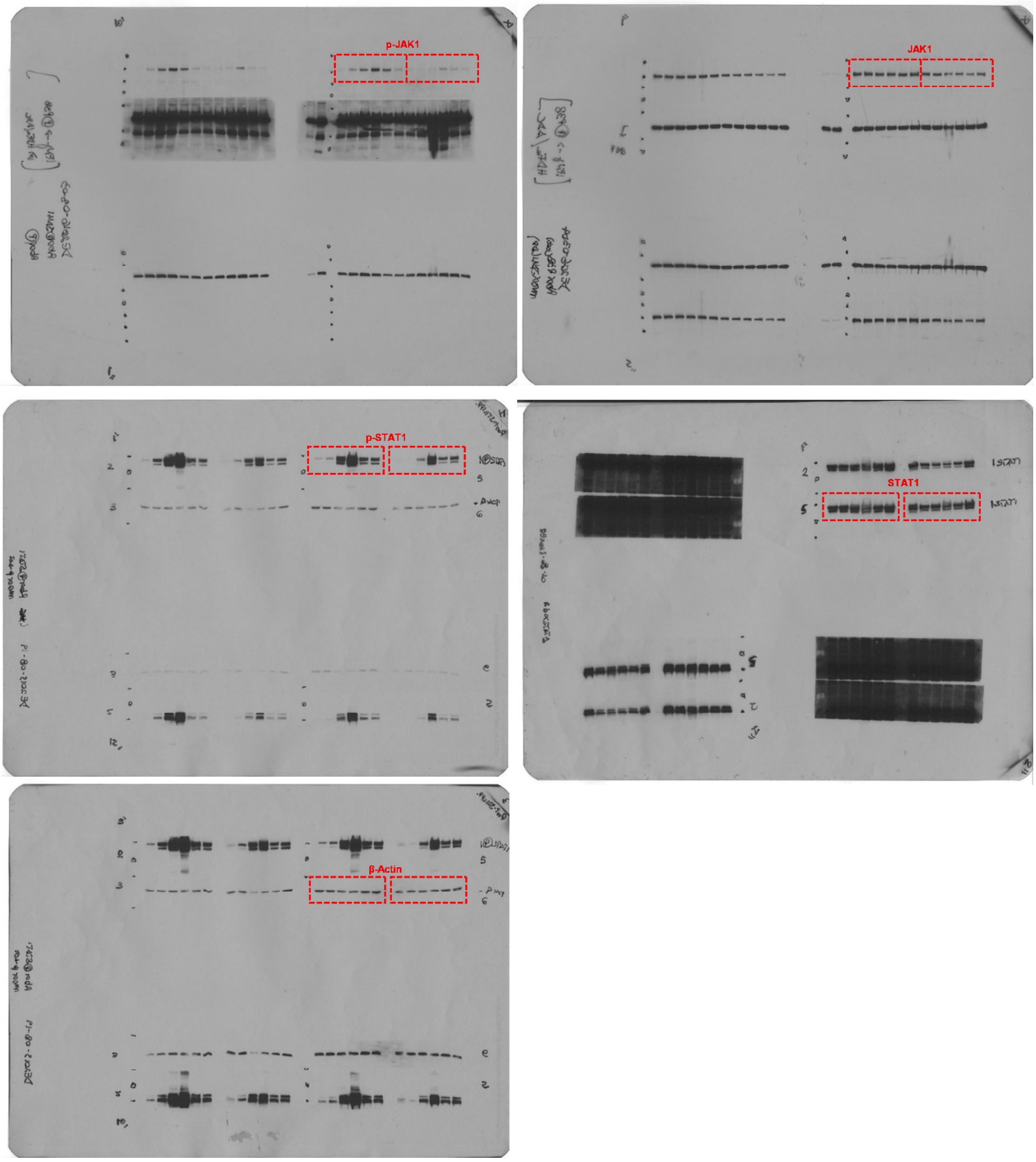

UNCROPPED SCANS – Fig. 6A

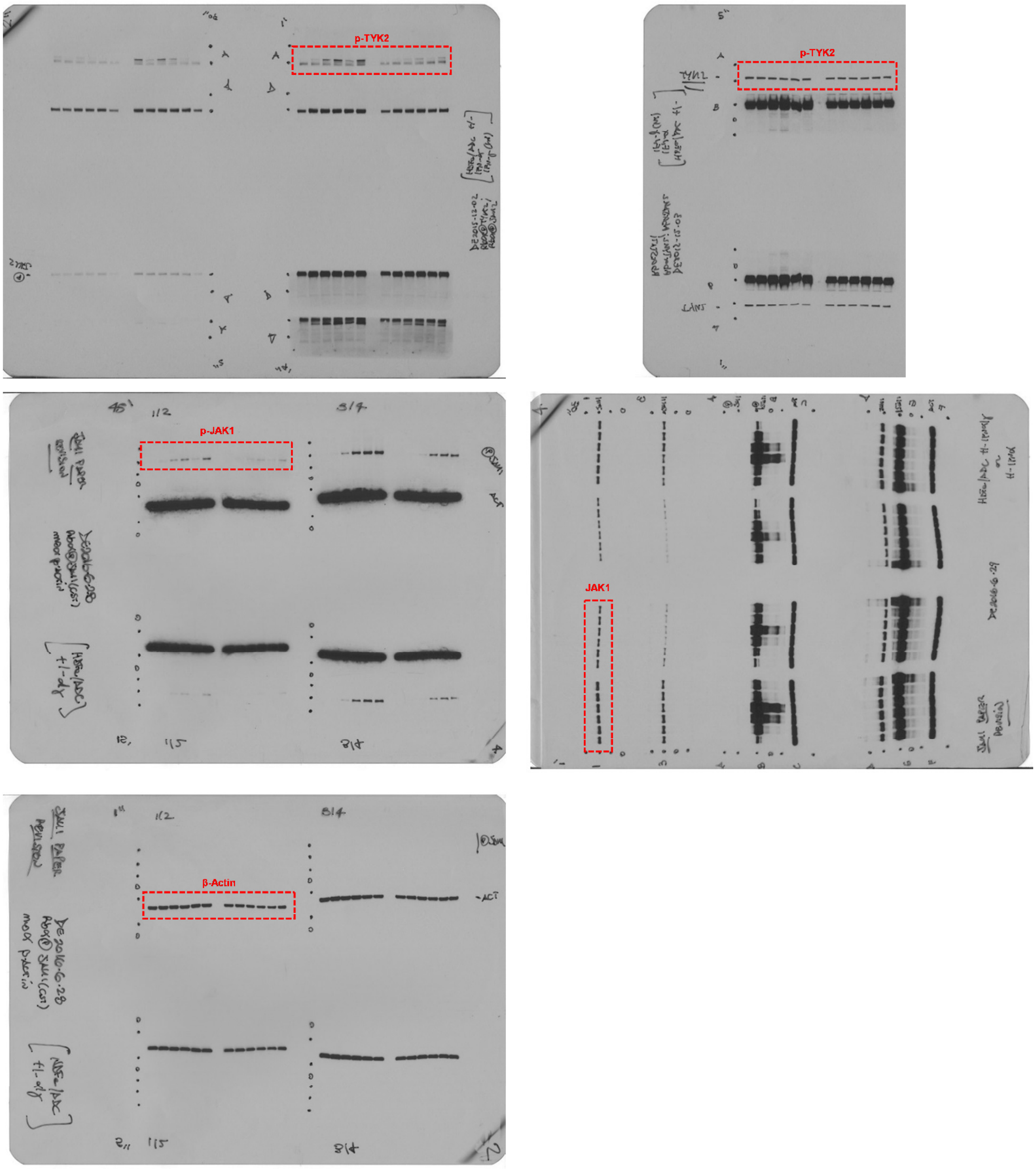

UNCROPPED SCANS – Fig. 6B

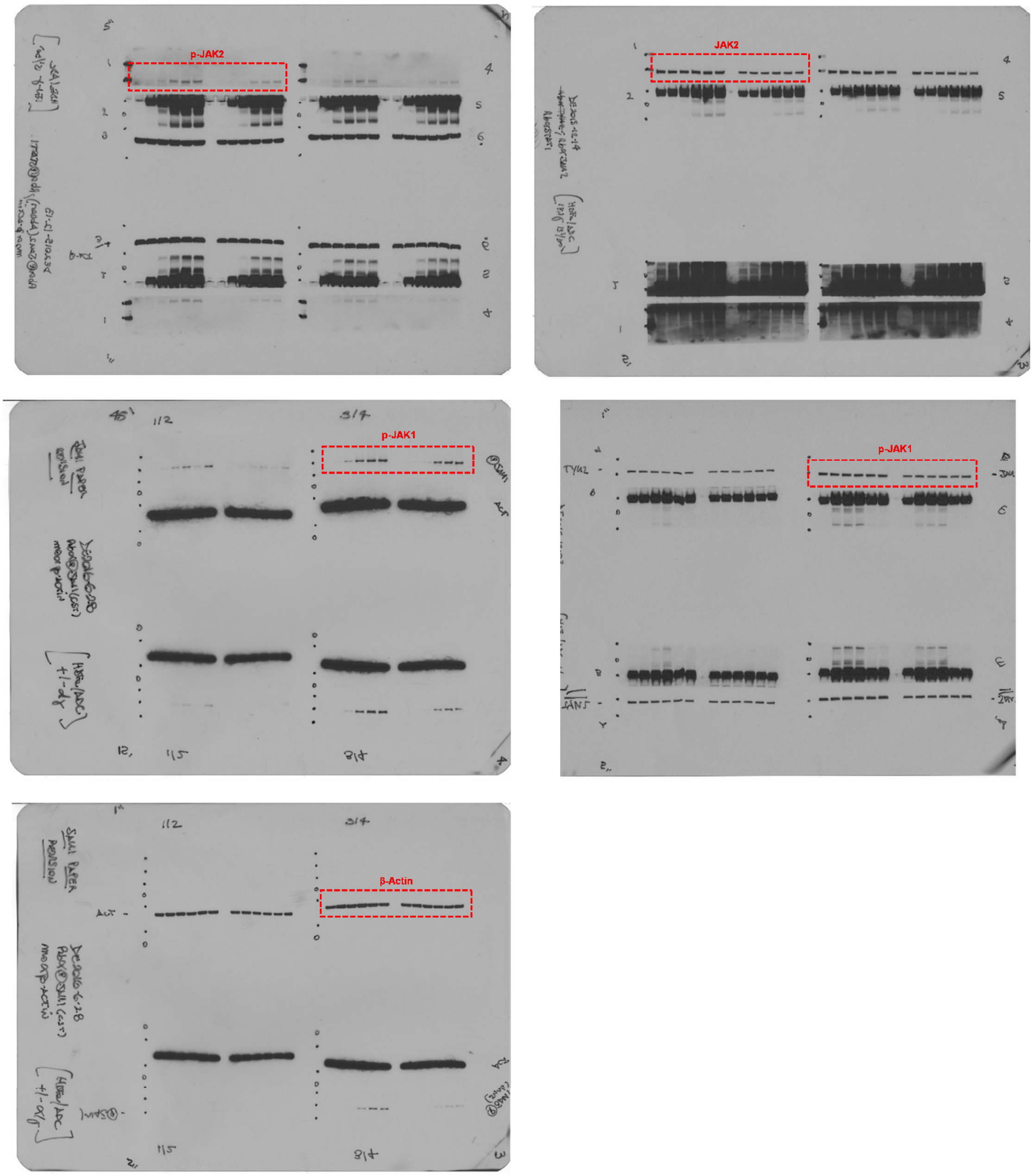

UNCROPPED SCANS – Fig. 7A

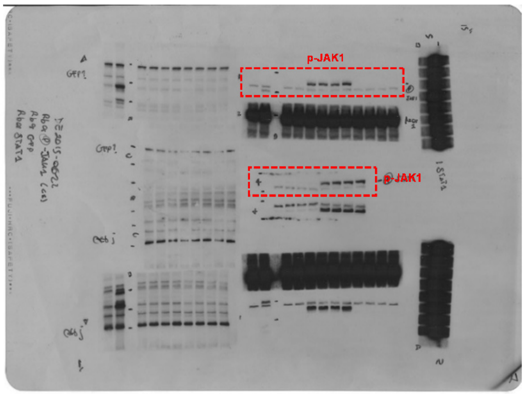

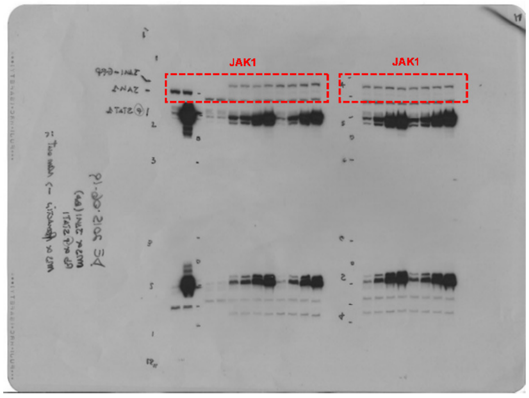

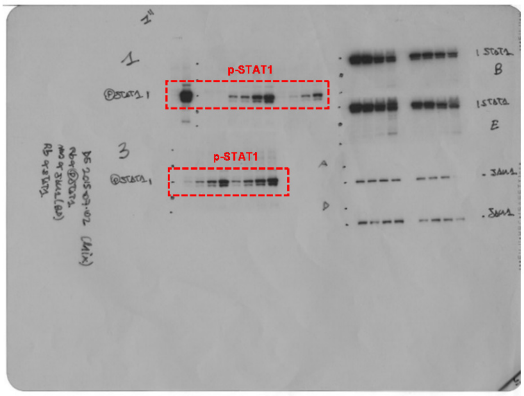

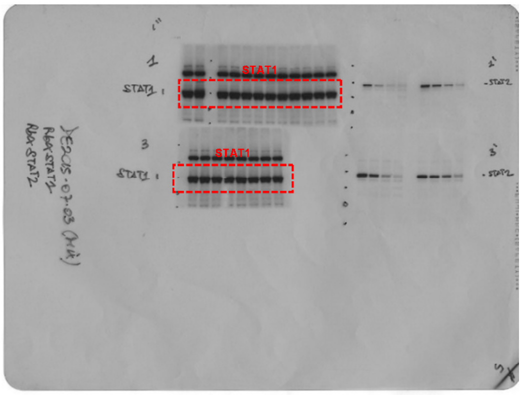

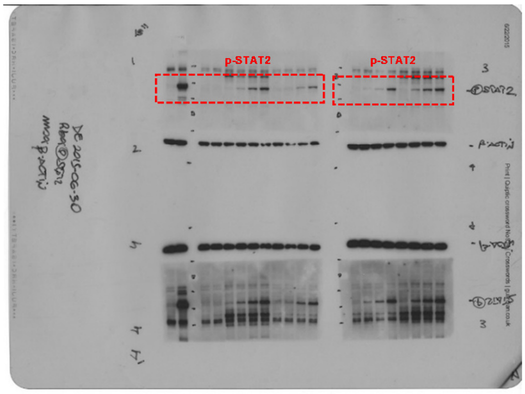

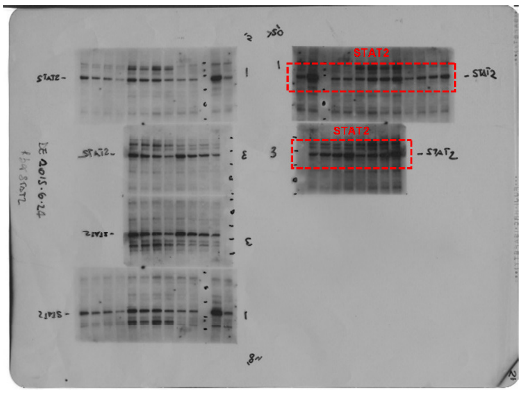

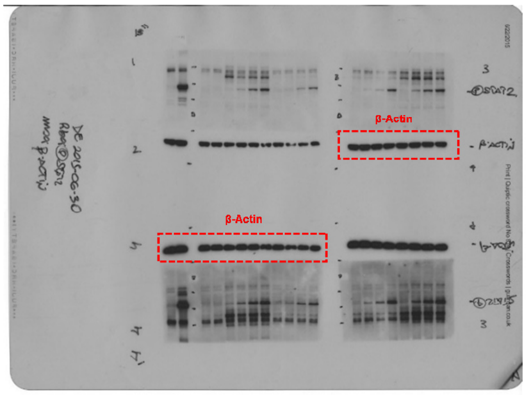

UNCROPPED SCANS – Fig. 7B

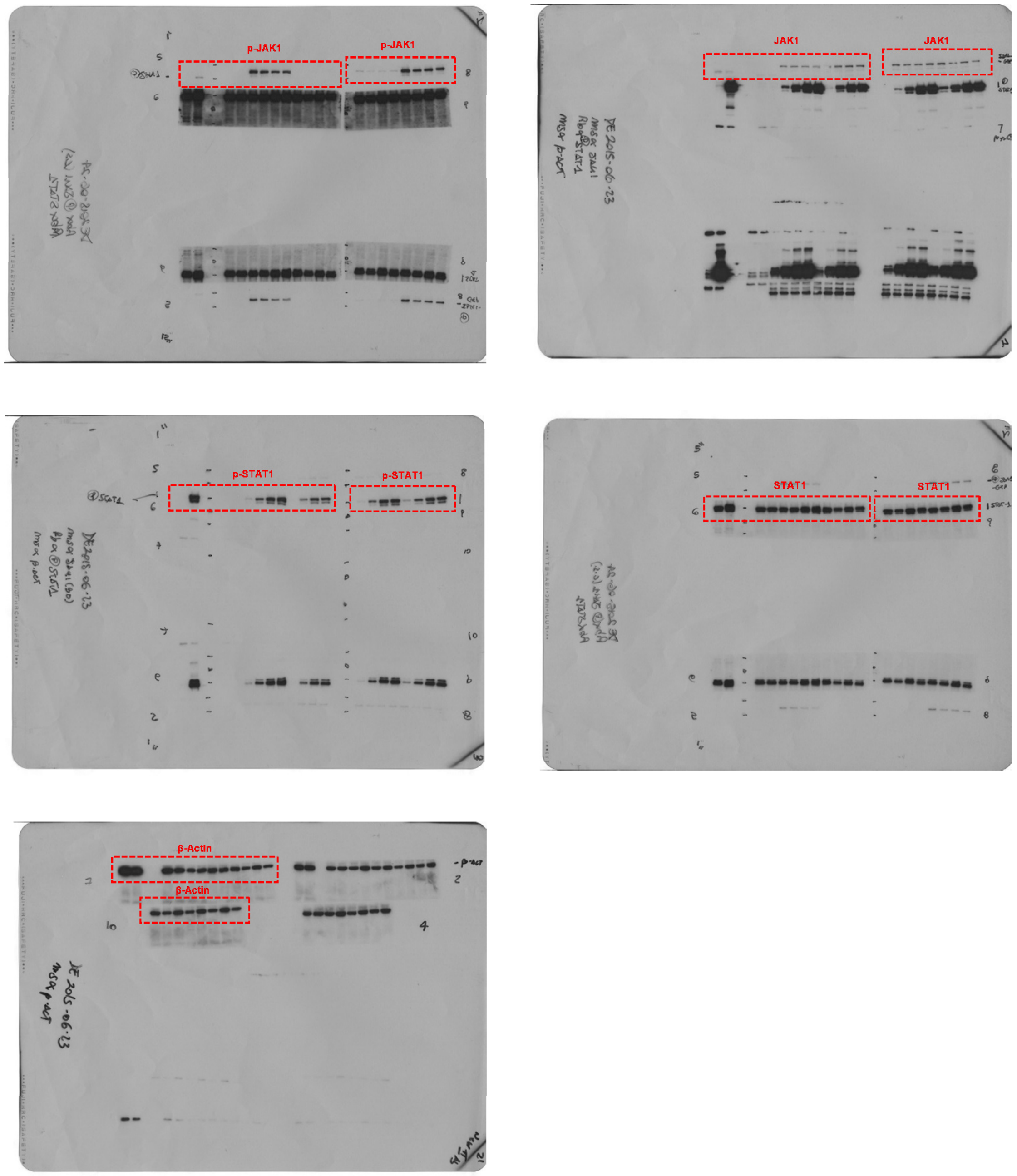

UNCROPPED SCANS – Fig. 7C

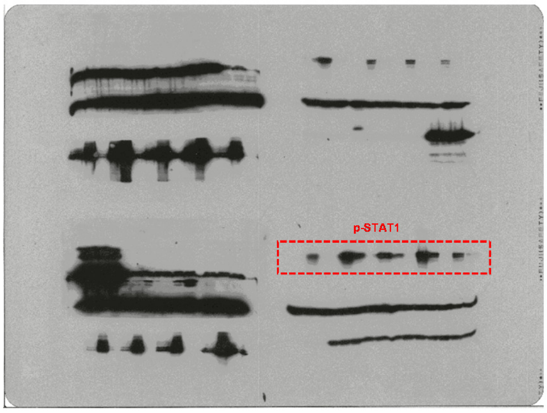

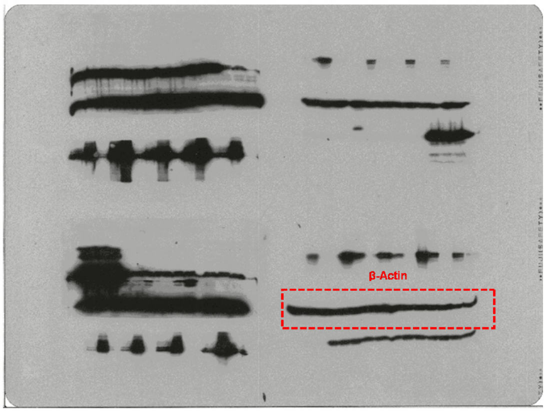

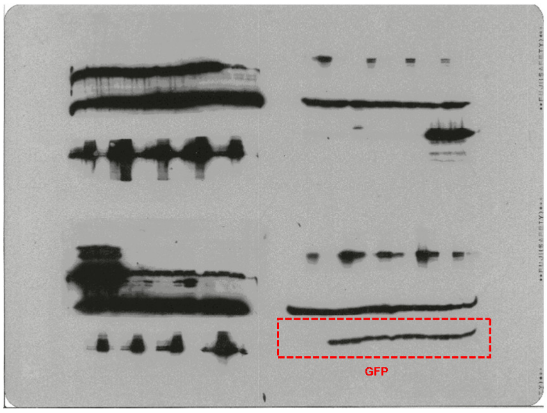

UNCROPPED SCANS – Fig. 8

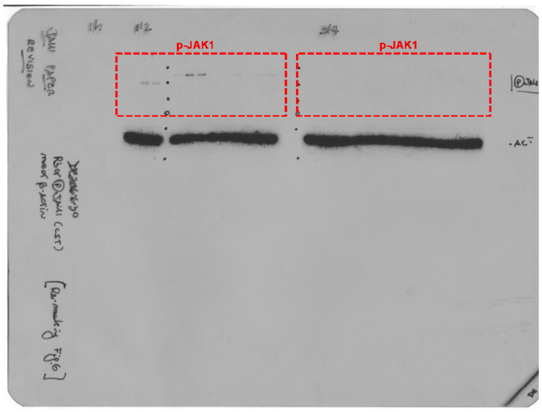

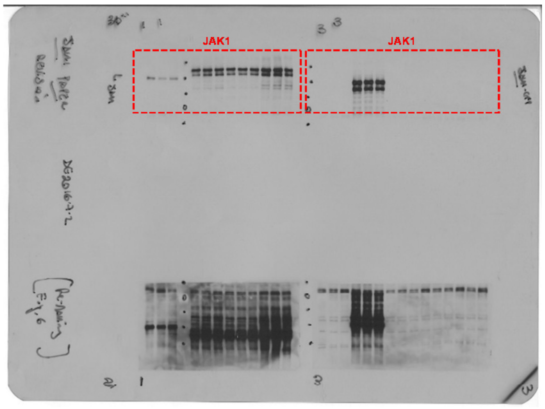

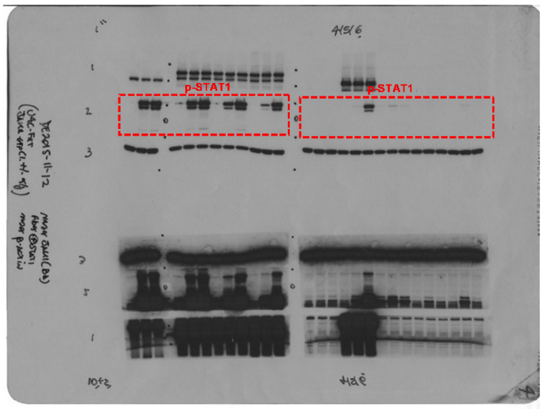

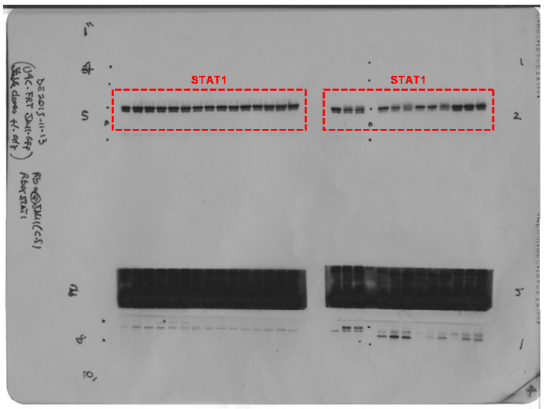

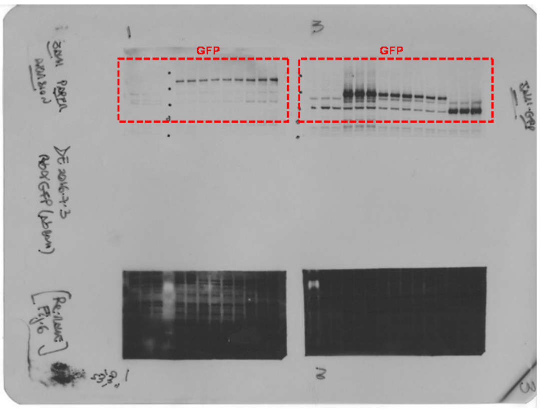

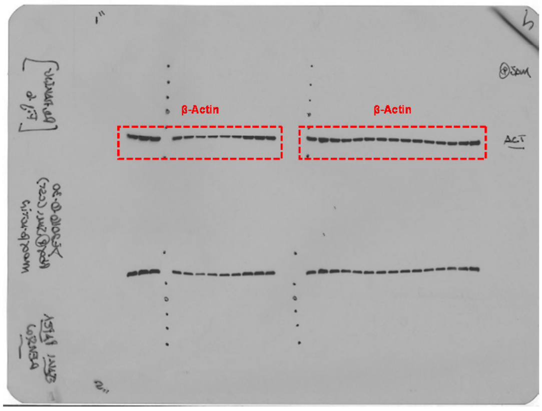

UNCROPPED SCANS – Suppl. Fig. 2A

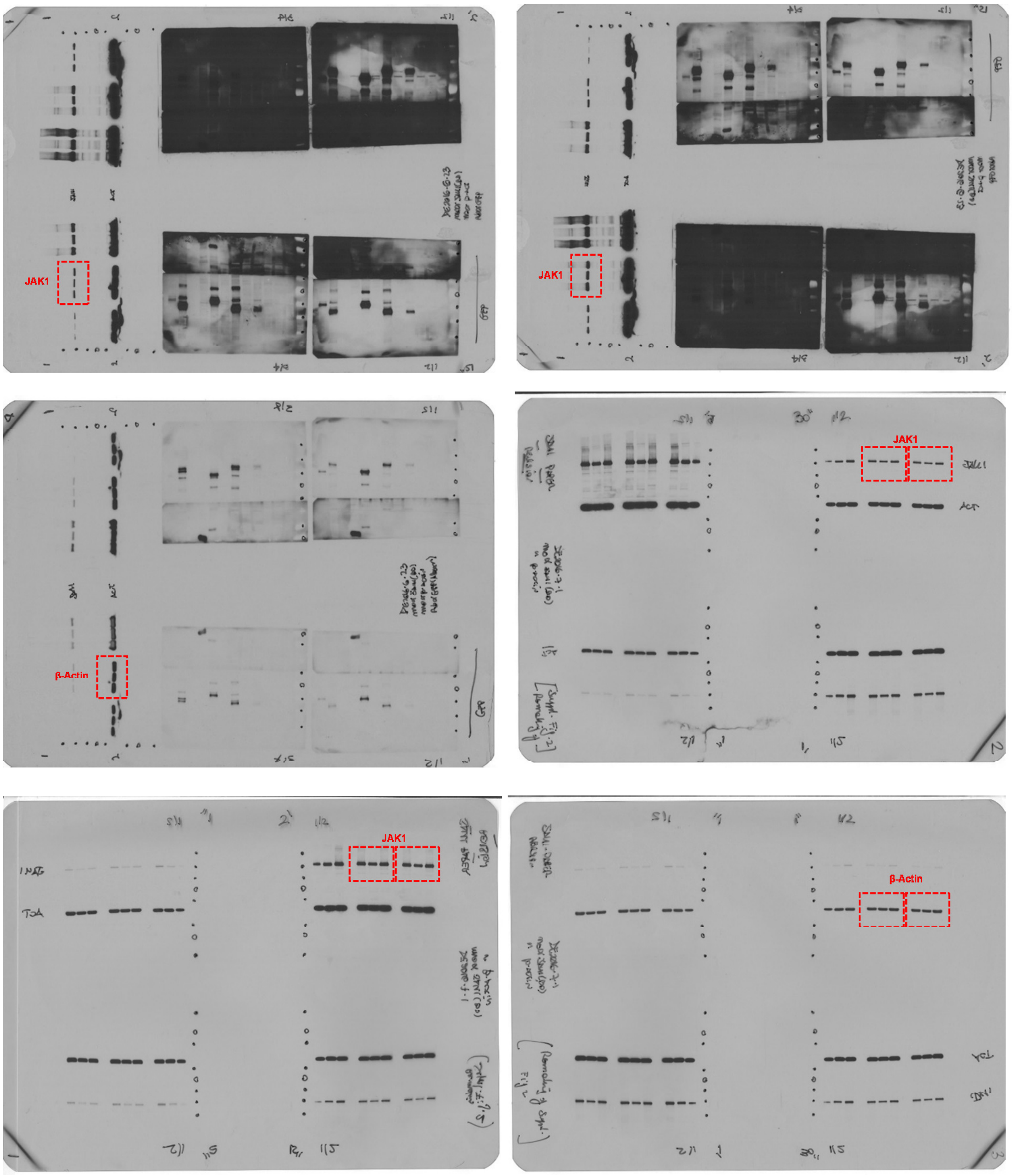

UNCROPPED SCANS – Suppl. Fig. 2B

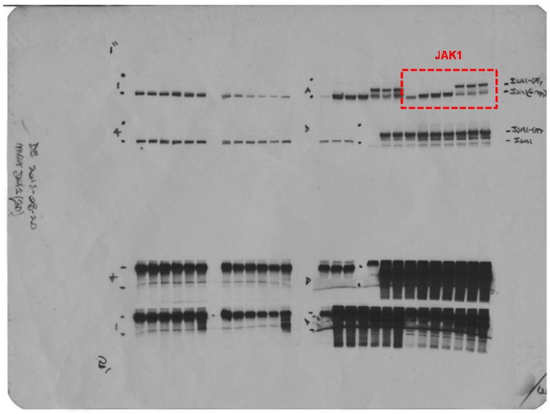

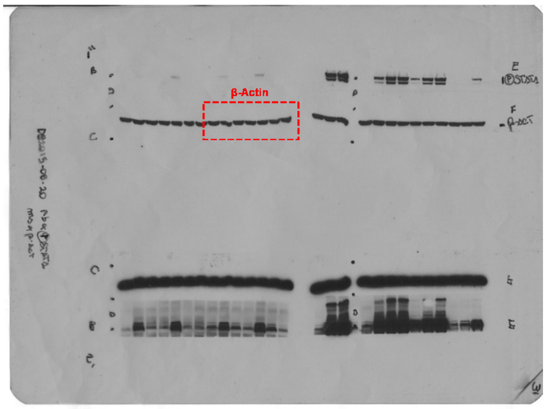

Supplement: Supplementary Information — Supplementary Figures and Supplementary Tables [file ncomms13992-s1.pdf]
